# Predicting location emotions of users considering multidimensional spatio-temporal dependencies

**DOI:** 10.3389/fpsyg.2025.1641623

**Published:** 2025-10-08

**Authors:** Wei Jiang, Yiming Wang, Xiaoqing Song, Xinyue Zheng, Xiang Liu, Yi Long, Zuo Wang, Ziran Wei

**Affiliations:** ^1^Anhui Normal University, Wuhu, China; ^2^Nanjing Normal University Key Laboratory of Virtual Geographic Environment Ministry of Education, Nanjing, China

**Keywords:** location emotion, spatio-temporal emotion prediction, multidimensional spatio-temporal dependencies, attention-based BiLSTM, graph embedding

## Abstract

Emotion has significant spatio-temporal characteristics, and predicting the spatio-temporal changes in emotion is an important premise for monitoring the emotional state of urban residents. Most prediction methods focus on the prediction of emotion in time series without considering the spatial properties of emotion. Based on geotagged image data on the Weibo platform from Shanghai, a user location emotion prediction method that considers multidimensional spatio-temporal dependencies between different emotional states is proposed in this paper. The method introduces the HiSpatialCluster algorithm to identify the users’ stay area. Then, the FaceReader algorithm is applied to determine the emotional quadrant of users from image data, and a graph embedding algorithm is employed to obtain the feature vector representing each stay area. Finally, an attention-based BiLSTM method is applied to construct the multidimensional spatio-temporal dependencies of emotion for prediction. Experiments on the Weibo dataset show that the prediction accuracy of location emotion reaches 75%, which is better than that of the single LSTM and CNN method. The results of this paper can not only deepen the understanding of the spatio-temporal variation patterns of emotion but also optimize location-based recommendation services.

## 1 Introduction

Emotion determines how people view the past, present, and future (Zhu and Gao, 2015). It is the distinguishing characteristic between humans and animals. Emotion is a psychological and physiological condition caused by the interaction of feelings, thoughts, and actions. It is distinguished by diversity and variability in spatio-temporal processes. Location emotion prediction refers to predicting the next spatial location of individuals or groups and their corresponding emotional states. Location emotion prediction is an emerging direction in the field of emotional computing and an important premise for monitoring the emotional state of urban residents.

Existing location prediction research is mainly based on big location data. The majority of these studies calculate the next location of people by fully investigating the spatio-temporal change pattern. In the early stage, studies mostly utilized statistical probability models to build spatio-temporal frameworks and then predicted the next location. For example, Ying et al. (2011) introduced a clustering-based model to predict the next location of a user’s movement. They analyze the geographical and semantic features of user trajectories to improve the prediction accuracy. Early methods have difficulty addressing spatio-temporal dependencies. Spatio-temporal dependencies refer to the relationship between data that are separated by different time periods and spatial distances. Deep learning methods provide effective approaches for constructing spatio-temporal dependencies. Some scholars have started to apply methods such as long short-term memory neural networks (LSTM) and conventional neural networks (CNN) to predict the next location. Zhang et al. (2019) proposed a CNN-LSTM network for location prediction; this method constructs spatio-temporal dependencies between trajectory data to predict the destination of the users. Existing studies show that a deep learning framework can effectively build spatio-temporal dependencies through time steps and gating mechanisms. Most existing prediction methods achieve a high level of accuracy. However, they still lack the consideration of the emotional attributes of users at the corresponding locations.

Emotion calculation is currently popular research in the field of computing. Most present emotion calculation approaches are based on machine learning. These methods can accurately extract the numerical features of the data and their contextual association. Yolcu et al. (2019) used multilayer convolutional neural networks to extract features of eyebrows, eyes and mouth regions in face images and achieved good prediction results on the RaFD dataset. Current methods can predict dynamic changes in emotional states in time series with a high accuracy. However, to our knowledge, there are no studies focusing on predicting emotion in different locations. Existing prediction methods have difficulty constructing multidimensional spatio-temporal dependencies, which is the key to location emotion prediction.

To address the above research problem, an attention-based BiLSTM method is proposed in this paper for predicting the target regions that users may visit and their corresponding emotional state. Compared with the existing methods, the proposed method achieves an accuracy of 75.21% in location emotion prediction. Our method can accurately and effectively predict location emotion. The results can not only deepen the understanding of the spatio-temporal change pattern of emotion but also provide an important theoretical basis for optimizing location-based personalized services.

## 2 Related works

### 2.1 Spatio-temporal location prediction

With the development of internet and mobile communication technology, the daily activities of urban residents have generated a large amount of spatio-temporal location data. Examples include social media data and cell phone data. The massive location data provide the possibility for prediction research. The existing location prediction methods can be broadly classified into shallow learning-based and machine learning-based prediction methods (Li et al., 2021).

Shallow learning methods mainly combine Markov chains (Douc et al., 2018) or Bayesian networks (Ben-Gal, 2008) to predict the location. Pavlovic et al. (2000) proposed a switching linear dynamical system model. The method is based on motion capture data to learn human motion characteristics for synthesis, classification and tracking of human motion to predict human trajectories. The results showed superiority over the traditional hidden Markov model (Eddy, 1996). To further improve the prediction accuracy, Kooij et al. (2014) developed a context-based dynamic Bayesian network model. The model integrates pedestrian situational awareness, situational criticality, and the spatial layout of the environment as potential states into the switching linear dynamical system. Thus, better prediction results than the switching linear dynamical system are obtained. Wang H. et al. (2019a) and Wang P. et al. (2019b) proposed a multi-order fusion Markov model to improve the algorithm. Based on the structural changes in the original trajectory, the model clusters the feature points to identify important locations and demonstrates superior predictive accuracy. However, the features extracted using shallow methods are insufficient. These methods also lack environmental semantic information and are complex to construct models. The final prediction results often deviate from the actual situation, and it is difficult to accurately predict spatio-temporal location.

In recent years, with the rapid development of machine learning, spatio-temporal location prediction methods based on deep learning have been rapidly developing. LSTM neural networks are often used to construct temporal dependencies to predict locations. Wang H. et al. (2019a) and Wang P. et al. (2019b) proposed a hybrid Markov model for location prediction. The model combines the Markov model and LSTM to capture the dependencies among location sequences to predict users’ locations. With the emergence of the attention mechanism, Karatzoglou et al. (2018) explored the effect of the attention mechanism on the model performance. The model uses the attention mechanism to extend the LSTM to predict locations, and the results show superiority over the traditional single-layer LSTM model. Xue et al. (2018) proposed a hierarchical LSTM model consisting of three scales. The model captures person-, social- and scene-level information using three LSTMs and obtains better prediction results. In addition to LSTM methods, CNNs are also basic neural networks that extract spatial features for location prediction. Niu et al. (2019) proposed the L-CNN model to predict taxi-passenger location. The model utilizes CNN to extract spatial dimension features and shows good performance with regards to the RMSE values. To further improve the prediction accuracy, Fan et al. (2018) adopted both CNN and bidirectional LSTM networks to predict the next location of vehicles. The method models periodic patterns and dynamic features of vehicle trajectories and shows superiority over several existing methods. Compared with shallow learning methods, deep learning-based models can effectively extract spatio-temporal-dependent information and achieve better prediction results. Substantial progress has been made in the field of multimodal spatio-temporal prediction, where models have integrated various data types like text, images, and location to improve prediction accuracy (Ho and Hui Lim, 2022; Halder et al., 2021). Han et al. (2014) notably enhanced location prediction accuracy by combining geographic reference information in text. Zhou et al. (2024) integrated multimodal spatio-temporal data, including traffic, text, and Points of Interest (POI), and proposed a comprehensive spatio-temporal prediction framework that significantly improves existing traffic prediction methods. Transformer-based models have become increasingly popular in location prediction due to their capacity to capture complex long-range dependencies (Sang et al., 2024; Saxena and Cao, 2022). Yu et al. (2020) proposed a Transformer-based approach for modeling crowd interactions, effectively addressing the problem of trajectory prediction. Additionally, Hong et al. (2022) developed a transformer decoder-based neural network to predict a user’s next location based on historical location, time, and travel mode.

However, existing models rarely take into account emotional information and lack in-depth exploration of spatio-temporal dependencies. The lack of spatio-temporal dependencies for emotion makes it difficult to predict spatio-temporal emotions. Therefore, capturing emotion dependencies is an urgent problem for current research.

### 2.2 Emotion calculation

With the rise of social media platforms such as Sina Weibo, Twitter, and Facebook, the daily online communication and sharing of urban residents has generated a large amount of social media data, which provides the possibility of emotion calculations. Existing emotion calculation methods broadly consist of two parts: emotion recognition and emotion prediction.

Traditional recognition methods mainly use manually designed features combined with classifiers for emotion recognition. Principal component analysis is one of the commonly used methods that can effectively extract the global features of the data and reduce the data dimensionality (Wold et al., 1987; Niu and Qiu, 2010). However, the recognition accuracy is limited, and it cannot make full use of the data information. For this reason, people started to extract features based on manually designed features for recognition. Such methods can improve recognition accuracy to a certain extent (Pantic and Stewart, 2007; Cootes et al., 1995). However, when key information is lost, the extracted features deviate, and the accuracy decreases. With the emergence of machine learning methods, people have started to apply them to extract features to improve the recognition accuracy. The optical flow method is often used to extract features, and Sun et al. (2019) proposed a multichannel deep spatio-temporal feature fusion neural network. This method uses optical flow to represent temporal features in static images, thus effectively recognizing face expressions in static images. Pan et al. (2019) extracted features from optical flow image sequences and designed a fully connected convergence layer for fusing different modal features. In addition to the optical flow method, an attention mechanism is also used to extract features. Sun et al. (2018) used CNN and a single-layer spatial attention mechanism to filter expression-related features. To further improve the accuracy, Mai et al. (2022) proposed a face expression recognition method based on a multiscale feature attention mechanism that uses two convolutional layers to extract information. The model introduces a channel attention mechanism to enhance the utilization of useful feature information. To construct the remote temporal association of features, LSTM is also used for emotion prediction. Liu and Wu (2020) proposed an emotion recognition model based on LSTM networks. The model further improves the accuracy of facial expression recognition by selecting multiple facial expression features for facial images.

Based on the correct recognition of emotion, classifiers can be combined or temporal associations of emotion can be constructed to predict future emotions. Nicolaou et al. (2012) proposed a novel output-associative relevance vector machine (OA-RVM) regression framework; the method employed a temporal window to learn temporal associations for emotion prediction in time series. Regarding text, word embedding are often used to extract semantic information from words and to analyze emotion. Thavareesan and Mahesan (2020) utilized Word2vec to represent each word and calculate their cosine similarity to classify different categories of emotions. BiLSTM is usually used to capture bidirectional long-time dependencies for emotion prediction. Li et al. (2020) used BiLSTM to extract sentence emotion and construct its contextual associations to predict text emotion. Zhang et al. (2020) introduced the whale optimization algorithm (WOA) to improve LSTM for the accurate prediction of public environmental emotions in time series. CNN is often employed to extract emotional features from images. Campos et al. (2017) studied the suitability of fine-tuning a CNN for visual feature extraction and used a linear layer to predict image emotion. Abdullah et al. (2018) proposed a coupled model of CNN and LSTM. It uses CNN to extract local features and LSTM to construct temporal correlations. The method achieves a higher prediction accuracy than CNN and LSTM.

Overall, these deep learning-based methods predict users’ emotional states by extracting feature factors of the data and constructing their mapping relationships with emotions. However, these methods are not accurate enough to construct the temporal correlations of emotion. They also rarely consider the location information and have difficulty constructing the multidimensional spatio-temporal dependencies of emotions. As a result, these methods cannot accurately predict the user’s spatio-temporal affective state.

## 3 Research methods and data sources

### 3.1 Experimental data and preprocessing

This study uses microblogs in Shanghai on the Weibo platform, and the original data include 748,790 microblogs with images posted by 267,737 users from 2017 to 2020. It is well known that Sina Weibo is the most popular microblog website in China. The platform has gained tremendous influence by significantly influencing the process of many real-world hot social events (Guan et al., 2014). The microblogs are collected based on the Weibo API, and each data sample includes the user’s ID, coordinates, post time, and the URL of the image, as shown in Table 1. We obtained the image from the microblog based on the URL.

**Table 1:** Microblog data.

Post time	*y*	*x*	ID	URL
Fri Jan 01 05:09:23 + 0800 2021	30.72**	121.35**	390***	http://wx1.sinaimg.cn/***.jpg
Fri Jan 01 03:16:56 + 0800 2021	30.70**	121.34**	189***	http://wx4.sinaimg.cn/***.jpg
Fri Jan 01 01:38:52 + 0800 2021	30.72**	121.33**	597***	http://wx1.sinaimg.cn/***.jpg
…	…	…	…	…
Fri Jan 01 01:11:50 + 0800 2021	30.72**	121.34**	293***	http://wx3.sinaimg.cn/***.jpg

**indicates hidden characters.

To create a reliable dataset, noise had to be removed. Microblog noise consists primarily of reposted microblogs, advertisements, and bot-posted microblogs. Geotagged microblogs cannot be reposted (Jiang et al., 2015). Therefore, geotagged microblogs have much less noise. During the preprocessing stage, we first obtained geotagged microblogs in Shanghai by filtering out coordinates. Most ads contain specific symbols, such as “【】.” Bot users often post duplicate messages for multiple days. We removed 42,167 microblogs containing these particular symbols. To avoid the sparsity of data on the experiment, we selected users who had posted at least 10 microblogs for 30 consecutive days as the experimental subjects based on existing social media research experience (Bao et al., 2021). Additionally, we strictly filtered the dataset to include only images containing human faces. The filtered dataset includes 206,881 microblogs from 11,996 users.

**Figure 1:**
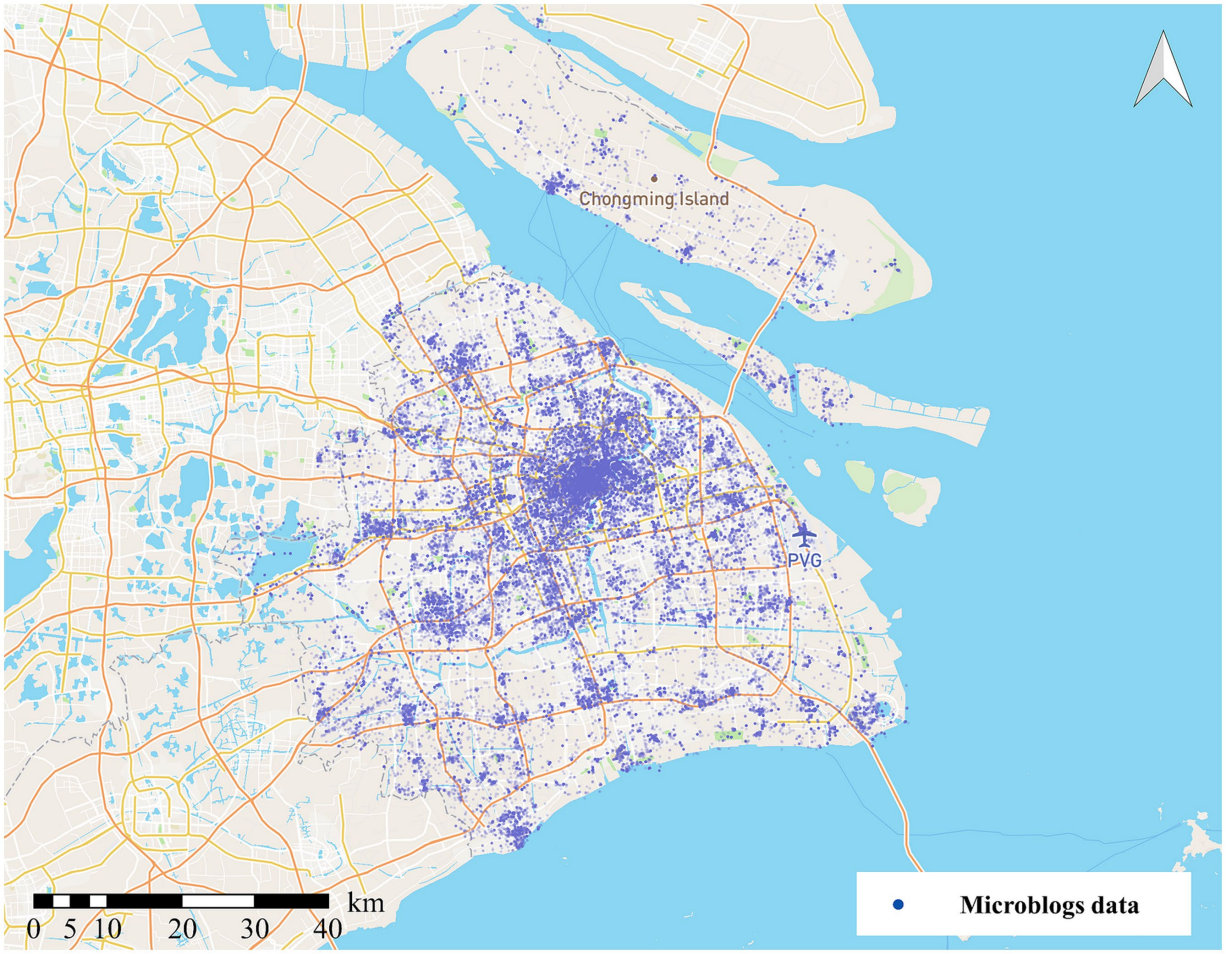
Microblog data distribution.

**Figure 2:**
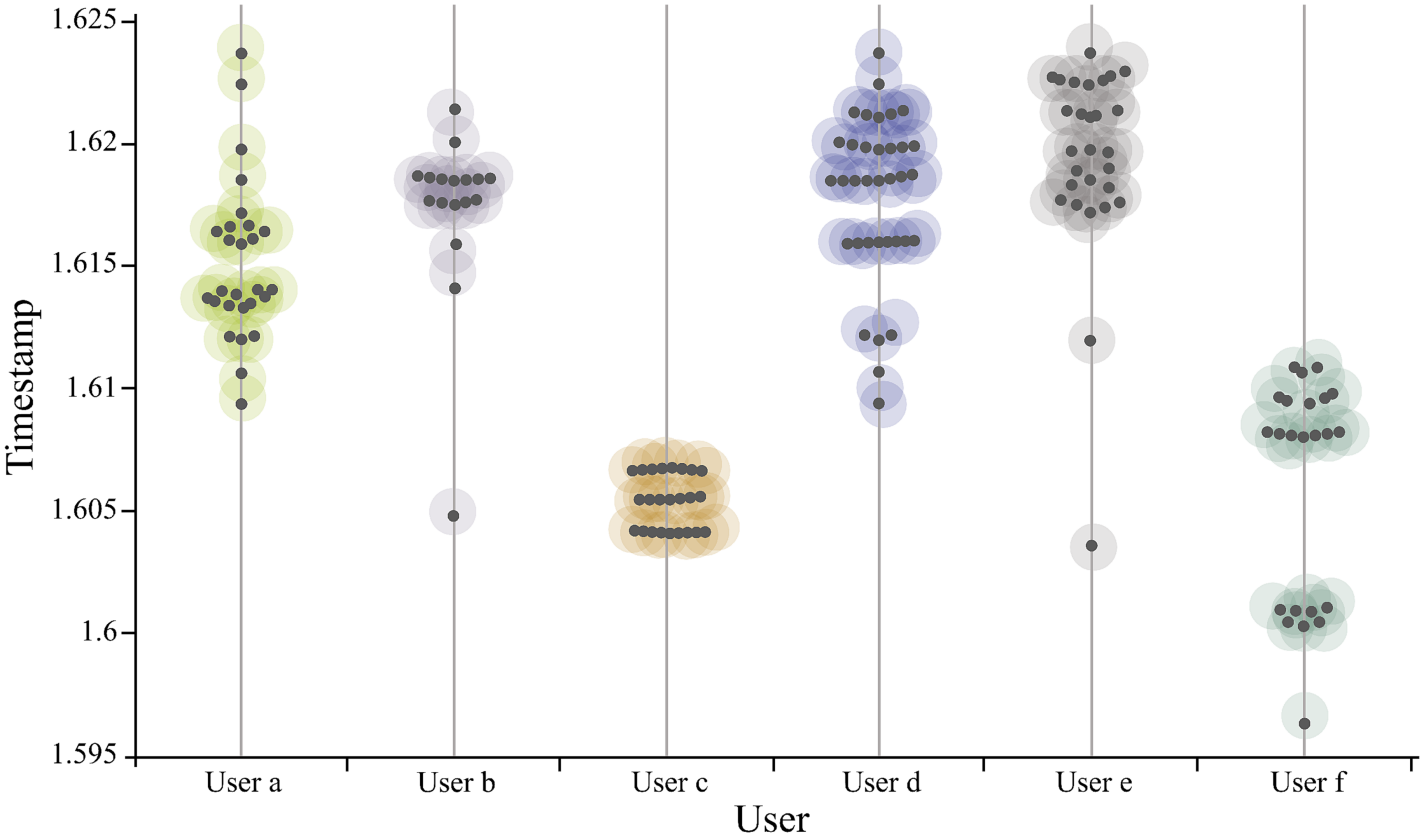
Check-in time distribution.

The spatial distribution of microblogs is shown in Figure 1. We discover that the spatial distribution of the microblogs is uneven. The urban centers concentrate the majority of data, whereas suburban locations post less data. Regarding the time dimension, Figure 2 illustrates microblog posting intervals, and clear temporal clustering can be observed. This research randomly chose six users and plotted their check-in time series distribution. There is a wide range of time between microblogs, ranging from a few days to several weeks.

### 3.2 Methodology

#### 3.2.1 Overall framework

**Figure 3:**
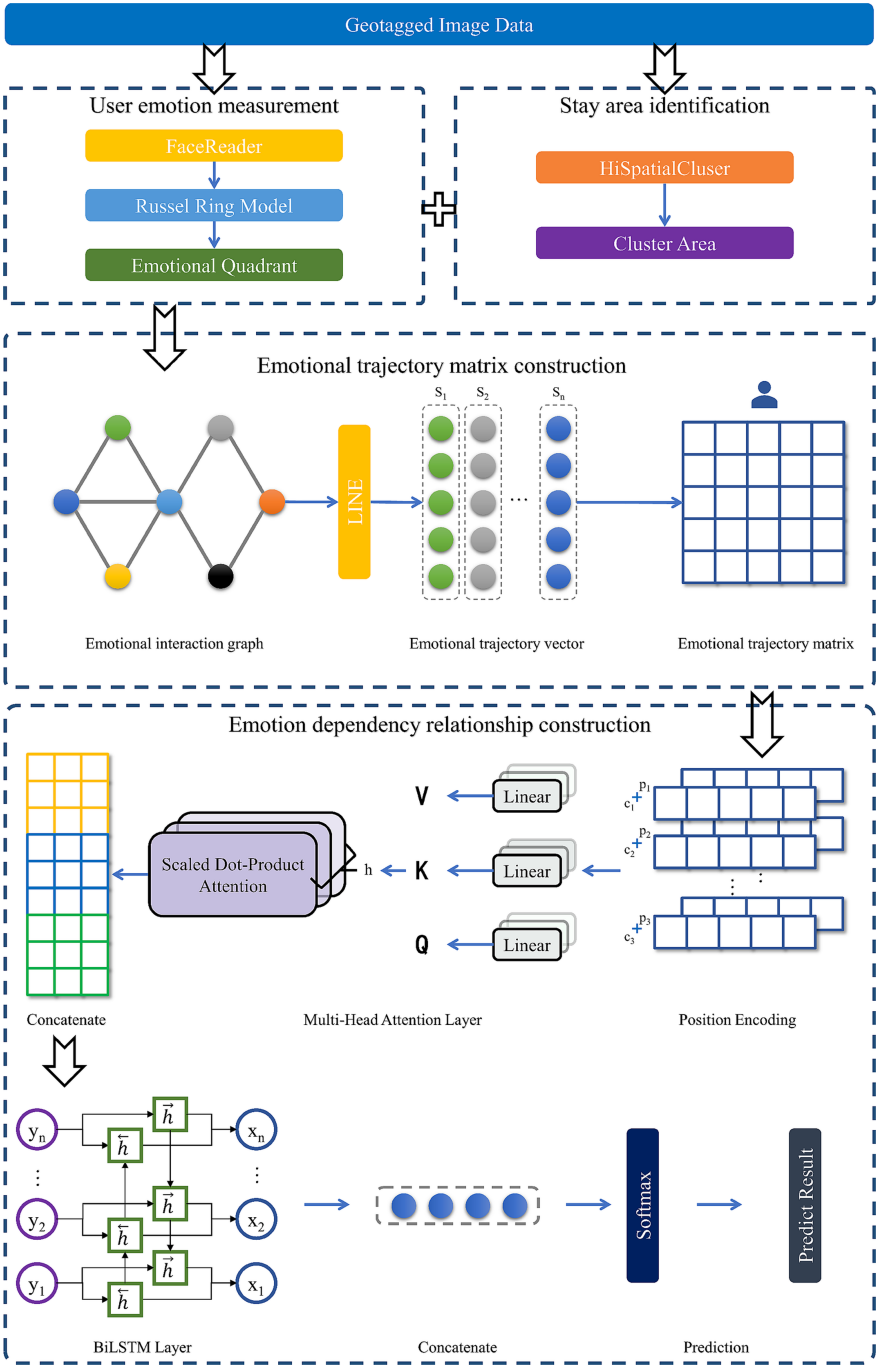
Flow chart of our proposed methodology.

The flow chart of the location emotion prediction model is illustrated in Figure 3. The method is divided into four parts: stay area identification, user emotion measurement, emotional trajectory matrix construction, and emotional dependency relationship construction. Initially, the clustering algorithm HiSpatialCluster is applied to determine possible stay areas. The images posted by Weibo users are analyzed using the FaceReader algorithm and Russell model for emotion measurement. Then, stay areas are abstracted into graph vertices to construct an emotional interaction graph, and each user’s trajectory matrix is calculated. Next, the original matrix is encoded according to the sequence. Time and space information are captured by a multi-head attention layer. BiLSTM layer outputs are concatenated with attention layer outputs to predict location and emotional quadrants.

#### 3.2.2 Stay area identification

Considering the sparsity of data, we partition the users’ staying area using the HiSpatialCluster clustering algorithm. HiSpatialCluster is an algorithm based on a fast search, density peak clustering and density connection filtering-based classification clustering. Adaptive clustering can be performed on massive point datasets with uneven spatial distributions of density. The algorithm divides check-in data into divisions of varying sizes based on data densities and balances the number of points inside each partition. Compared with existing clustering methods, such as DBSCAN (Ester et al., 1996) and K-Means (Bock, 2007), HiSpatialCluster has better robustness. It can effectively filter noise points, quickly cluster massive data, and quickly reach convergence. In this study, we adopt HiSpatialCluster for clustering large-scale geotagged social media data. Compared to DBSCAN and KMeans, HiSpatialCluster offers distinct advantages in handling datasets with uneven density and regional heterogeneity. Previous studies (Chen et al., 2018; Bao et al., 2021) have empirically demonstrated that HiSpatialCluster outperforms DBSCAN and KMeans in clustering large spatial point datasets, particularly those characterized by varying densities. Both studies emphasize that HiSpatialCluster not only improves the accuracy of delineating meaningful spatial regions but also maintains high computational efficiency when applied to large-scale datasets. Given the similarity of our dataset to those used in prior studies in terms of platform, scale, spatial distribution, and research objective, we adopted HiSpatialCluster to perform the clustering analysis in this study. After clustering the data and generating the clustered areas, the emotion attributes can be attached to the clustered areas.

#### 3.2.3 User emotion measurement

**Figure 4:**
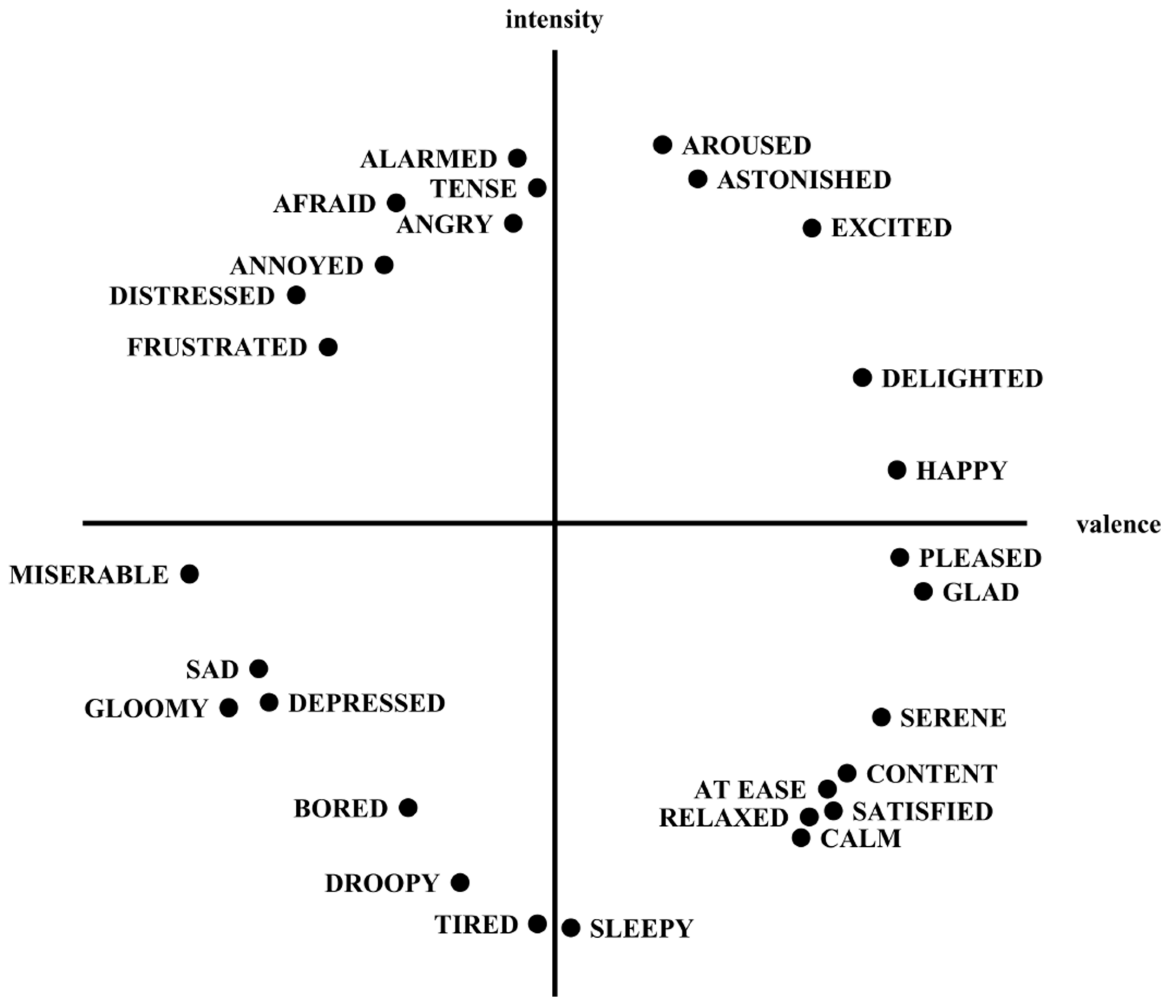
A circumplex model of emotions.

In this paper, the Russell ring model (Russell, 1980) and FaceReader algorithm are combined to obtain the emotional quadrants and measure the emotion of expressions in images. Russell’s ring model is an emotion classification method, as shown in Figure 4. It divides emotion into two dimensions: valence and intensity. The valence indicates the degree of pleasure of emotion, and the intensity indicates the level of emotion. As a result, we divided the emotions into four quadrants. The first quadrant is the high valence value-high intensity state, the second quadrant is the low valence value-high intensity state, the third quadrant is the low valence value-low intensity state, and the fourth quadrant is the high valence value-low intensity state. Thus, four significantly different emotional states can be distinguished corresponding to four emotional quadrants. All emotional emotions can be represented as a point in a two-dimensional coordinate system. Existing studies on emotion classification in images and text typically focus on differentiating between positive and negative polarity (Liu et al., 2023). Our approach extends this framework by employing a Russell ring model to segment emotions into four distinct quadrants, offering a more comprehensive and nuanced understanding of a user’s emotional state. FaceReader can recognize the valence and intensity of facial expressions in images. It is a high-precision emotion recognition method based on a deep learning framework. FaceReader is proficient in accurately recognizing emotions based on facial expressions and effectively monitoring users’ emotional states (Terzis et al., 2010). The Viola-Jones algorithm is applied to find the location of faces in images based on more than 500 key points of the face, and a deep neural network is utilized to determine the emotional state of the face. The output of FaceReader is the intensity and valence of seven-dimensional emotions (happy, sad, angry, surprised, fearful, disgusted, and neutral). Although FaceReader outputs seven discrete emotions, some of these expressions (e.g., angry and disgusted) share highly similar facial features, limiting the algorithm’s ability to distinguish similar expressions. Directly using the seven categories as label may therefore propagate classification errors into subsequent analyses. To enhance prediction reliability and ensure clearer differentiation, we reclassified the seven emotions into four emotion quadrants based on the Russell ring model (Cittadini et al., 2023).

FaceReader analyzes facial expressions and outputs emotion data that corresponds to a point on the Russell ring model. The coordinates of this point determine the corresponding valence ( $x_{i}$ ) and intensity ( $y_{i}$ ) of the emotion. For each image, the ${\text{Emotion}}_{i}$ is then represented using Equation 1:


(1) $${\text{Emotion}}_{i}=(x_{i},y_{i})$$

where $x_{i}$ and $y_{i}$ denote the valence and the intensity of image i, respectively.

For a Sina Weibo post containing n images, the ${\text{Emotion}}_{i}$ values for all images are aggregated, and the resulting composite emotion is classified into one of four emotional quadrants. These quadrants are defined as follows: the first quadrant corresponds to high valence and high intensity; the second quadrant corresponds to low valence and high intensity; the third quadrant corresponds to low valence and low intensity; and the fourth quadrant corresponds to high valence and low intensity. The dimensional representation of emotion, denoted as dimension , is a two-dimensional array derived from the aggregation of the ${\text{Emotion}}_{i}$ values, as indicated by Equation 2. The Dimension array’s coordinates are then used to determine the corresponding emotional quadrant.


(2) $$\text{dime}\text{nsion}=\sum_{i=1}^{n}\text{Emotio}n_{i}$$

The coordinates of the aggregated emotion ( dimension ) are mapped to the four quadrants of the Russell ring model, where each quadrant reflects a distinct combination of valence and intensity. The emotional quadrant allows us to identify the emotional state of a user in a particular spatio-temporal area. By acquiring the emotion quadrant of images, it can provide data support for the construction of emotional trajectory matrices.

#### 3.2.4 Emotional trajectory matrix construction

The construction of the emotional trajectory matrix consists of two steps: the construction of the emotional graph and the calculation of the emotional trajectory vector.

**Figure 5:**
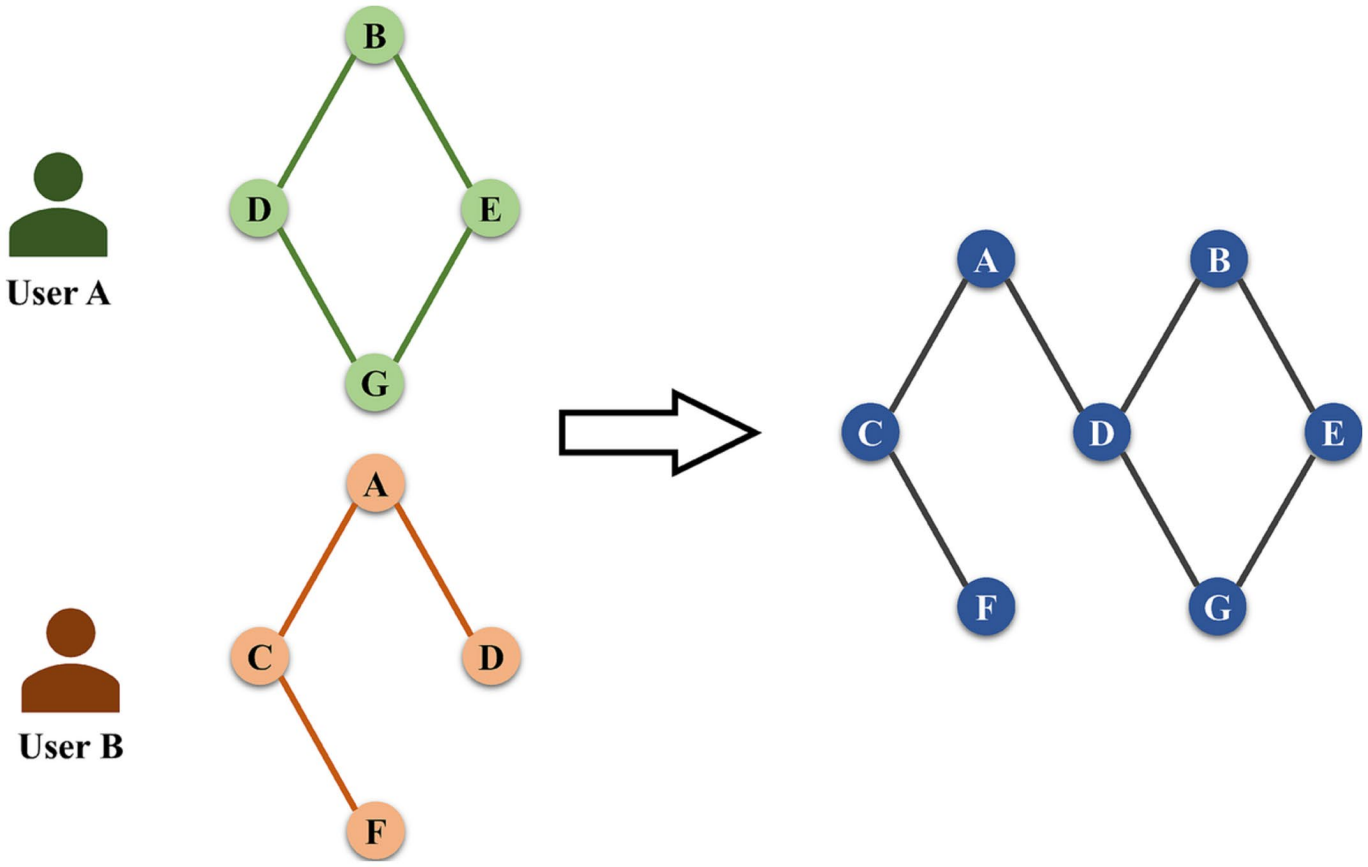
Emotional interaction graph construction.

*Emotional interaction graph construction.* We use the emotional interaction graph to quantify the travel preferences and the emotional state in each stay area, as shown in Figure 5. Based on the users’ stay area and the emotional quadrant, the emotional interaction graph is developed using graph theory. In addition, the graph quantifies the interaction relationship of the users’ emotions between each stay area. There are *n* nodes in the emotional interaction graph, each of which contains two properties: the stay area and the emotion quadrant, as shown in Equation 3:


(3) $$\begin{array}{c} V=\{(C_{1},E_{1}\;),(C_{2},E_{2}\;),(C_{3},E_{3})\ldots (C_{n},E_{n}\;)\} \end{array}$$

where *C_i_* denotes the stay area, and *E_i_* represents the emotion quadrant. After constructing the emotional interaction graph, we use *U_k_* to denote the *k_th_* user trajectory as shown in Equation 4.


(4) $$\begin{array}{c} U_{k}=\{V_{1k},t_{1},V_{2k},t_{2}\ldots V_{\mathrm{mk}},t_{m}\;\}(t_{1}<t_{2}<t_{3}\ldots <t_{m}) \end{array}$$

where *V_k_* is the corresponding node in the emotional interaction graph, and *t_m_* is the time of the *m_th_* microblog posting.

In this paper, we define the weights of edges using the function g(·). The weights between two adjacent vertices *V_i_^k^* and *V_j_^k^* are computed as follows:


(5) $$\begin{array}{c} g(U_{V_{i}^{k},V_{j}^{k}}^{k})=\frac{1}{1+e^{-\frac{\text{Tmax}}{\alpha (t_{j}-t_{i})}}} \end{array}$$

where *α* is used to regulate the weight of time intervals. Based on the experience of previous studies, *α* = 10 is set in this study as applicable to the dataset. *T_max_* is the maximum time interval for the *k_th_* user to post a microblog. If there are adjacent nodes *V_i_* and *V_j_* that are visited by multiple users, the weights corresponding to all users’ sequences are first calculated separately using Equation 5. Then, Equation 6 is used to calculate the weights of the edges between *V_i_* and *V_j_* in the whole emotional interaction graph.


(6) $$\begin{array}{c} \text{Edge}=(V_{i},V_{j})=\Sigma_{k=1}^{s}g(U_{V_{i}^{k},V_{j}^{k}}^{k})\text{if}\exists V_{i}^{k},V_{j}^{k}:V_{i}=V_{i}^{k},V_{j}=V_{j}^{k} \end{array}$$

where *V* is the set of vertices, and *E_VV_* contains all weighted edges.

**Figure 6:**
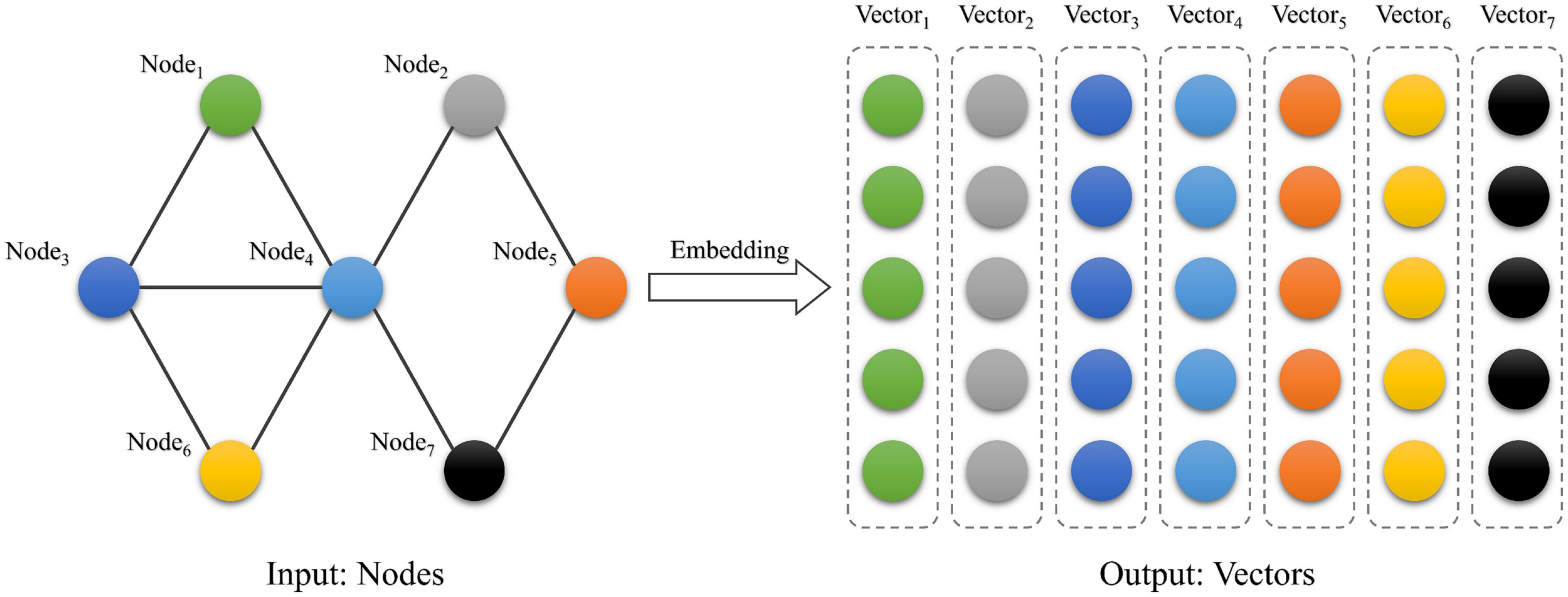
Graph embedding.

*Emotional trajectory vector calculation.* To transform the graph structure into vectors for model computation, this study constructs emotion trajectory vectors based on the LINE algorithm and builds the users’ emotional trajectory matrix. LINE is an algorithm for graph embedding (Tang et al., 2015) that generates a low-dimensional vector representation of the graph’s vertices. Liu et al. (2023) modeled and integrated user behavior and social influence by constructing a social behavior graph. Graph embedding is employed to transform social networks into learnable low-dimensional representations, thereby capturing latent social relationships. Similarly, our study develops emotion interaction graph to integrate users’ spatial and emotional information. The LINE method is applied to generate embedding vectors that represent the spatio-temporal correlations of user emotions. Several approaches for graph embedding have been proposed, including DeepWalk (Perozzi et al., 2014), node2vec (Grover and Leskovec, 2016) and LINE. While DeepWalk and Node2Vec effectively capture local (first-order) neighborhood structures, their ability to represent higher-order proximities is limited, making it difficult to model indirect dependencies (Tang et al., 2015). In emotional trajectory analysis, such indirect relationships are essential, as emotional influence often propagates through multiple intermediaries rather than being confined to direct interactions. By explicitly preserving both first-order and second-order proximities, LINE provides a more comprehensive representation of influence patterns across the network. Compared with traditional graph embedding algorithms, the LINE algorithm captures both first- and second-order proximities. The ability to model second-order proximity enables LINE to represent latent, indirect connections between nodes. This provides a more nuanced representation of spatio-temporal emotional interactions, where immediate links may not fully explain the underlying dynamics. Meanwhile, LINE proposes an edge sampling algorithm to improve stability and is more suitable for embedding large-scale graphs. Figure 6 shows the LINE embedding process.

The vertex vectors generated from the LINE algorithm can effectively reflect the potential spatio-temporal correlation between the emotions of various stay areas. In the graph structure, highly correlated vertices correspond to larger edge weights. Even if two vertices are not directly connected, they may share the same vertices and thus can be considered to have similar states in the graph. This condition is similar to the second-order approximation of LINE. Therefore, LINE is used to process the spatio-temporal emotional interaction graph and construct the low-dimensional vector for each stay area *C_i_*. In the graph, each stay area *C_i_* plays two roles, its own and a specific context. There are two types of distributions that can be defined for these two kinds of roles: the conditional distribution ${\hat{p}}_{i}$ and the empirical distribution ${\hat{p}}_{2}$ . The objective of LINE is to minimize the distance between the two distributions using Equation 7:


(7) $$\begin{array}{c} O_{2}=-\sum_{i\in V}\lambda_{i}\mathrm{KL}(p_{i},{\hat{p}}_{i}) \end{array}$$

KL(∙, ∙) is the KL scatter between the two distributions. *λ_i_* is the out-degree of *C_i_* that represents the importance of the vertices in the whole graph. The low-dimensional vector representation $\vec{u_{i}}$ of each stay area *C_i_* can be obtained by minimizing O_2_.

**Figure 7:**
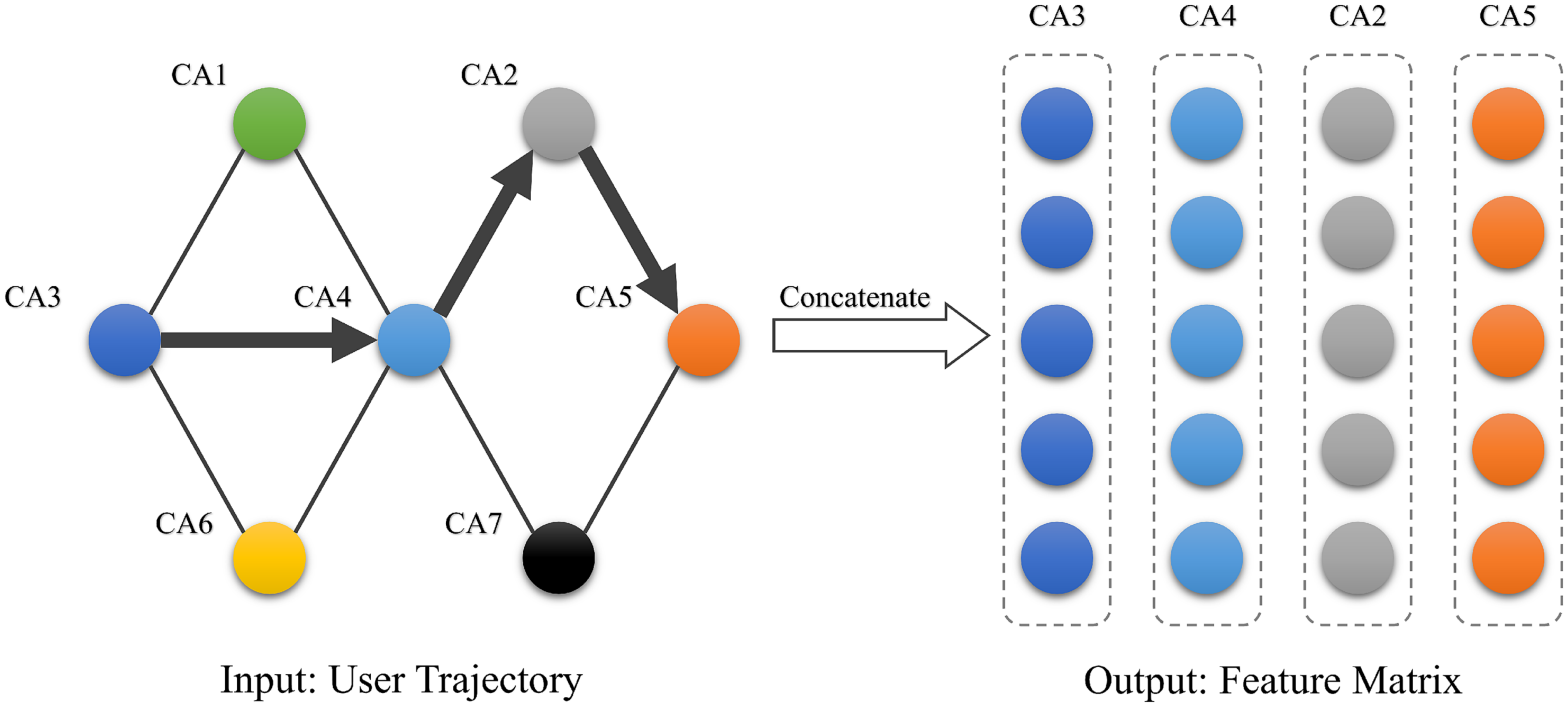
User emotion matrix.

After obtaining the vectors representing each stay area, the vectors are sequentially stitched in chronological order based on their historical temporal information. As shown in Figure 7, we obtain a matrix of users’ spatio-temporal emotion trajectories as the input to the prediction model. The trajectory matrix of the *k_th_* user can be expressed as $F_{k}=[{\vec{u}}_{1}^{k},{\vec{u}}_{2}^{k},{\vec{u}}_{3}^{k}\cdots {\vec{u}}_{m-1}^{k}]$ .

#### 3.2.5 Emotion dependency relationship construction

The construction of multidimensional spatio-temporal dependencies of emotions is divided into two parts: the encoding of location and the construction of dependencies. Building emotion spatio-temporal dependencies is an important part of extracting the interaction information from the trajectory matrix. By constructing the spatio-temporal dependencies of the emotion trajectory matrix, we can more accurately capture the dependencies of the matrix. Furthermore, it allows us to fully investigate the spatio-temporal emotion interaction information of users.

We encode the emotion trajectory vectors to capture the contextual information of the emotion trajectory vector. Then, we investigate spatio-temporal information from the emotion trajectory vectors. For each vector in the matrix, encoding vectors are generated based on the vector’s position in the sequence and its dimensionality. Next, it is correspondingly added to the original emotion trajectory vectors. The position encoding is calculated using Equations 8 and 9.


(8) $$\begin{array}{c} PE_{\left(\mathrm{pos},2i\right)}=\sin(\mathrm{pos}/10000^{2i/d}) \end{array}$$

(9) $$\begin{array}{c} PE_{\left(\mathrm{pos},2i+1\right)}=\cos(\mathrm{pos}/10000^{2i/d}) \end{array}$$

where *pos* is the position of the emotion trajectory vector in the emotion trajectory matrix, and *d* is the dimension of the emotion vector.

**Figure 8:**
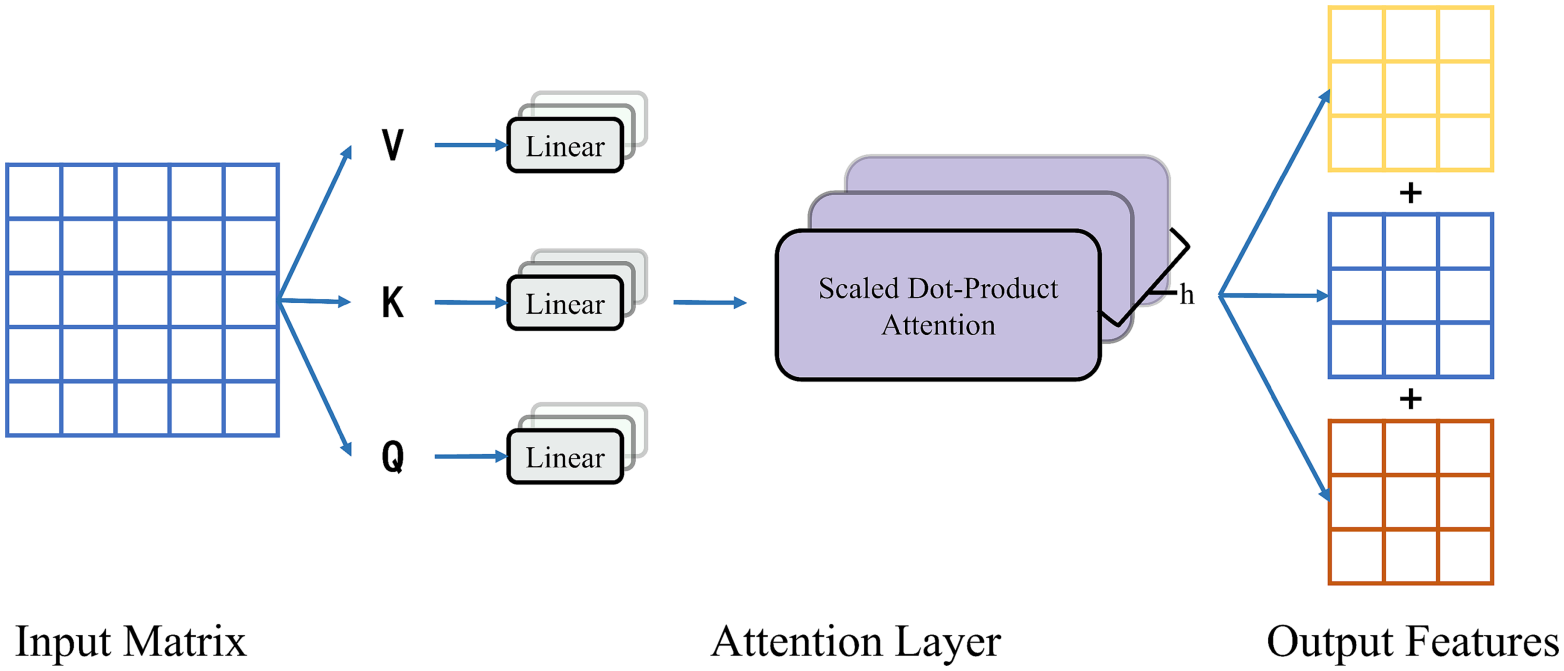
Multi-head attentional mechanism.

After incorporating the contextual information, the multidimensional spatio-temporal dependencies of emotions are constructed based on the attention-based BiLSTM model. The main idea of the attention mechanism is to assign different weights to each part of the input and allocate varying degrees of attention according to the weight size during the decoding process. Unlike traditional RNNs, the attention mechanism allows the model to focus on the parts that contribute to prediction and ignore the irrelevant parts. It is possible to dynamically adjust the weight size of the model based on the input of the model to make more accurate judgments. To obtain multidimensional features, a multiheaded attention mechanism integrates multiple self-attentive mechanisms. As shown in Figure 8, the weights of each part are reassigned by softmax normalization after dynamically calculating the similarity between each vector. Finally, weighting and summing are performed to produce the new matrix. The initial three matrices K, Q, and V are scaled by the dot product between them and normalized by the softmax layer to extract the spatial and temporal dependence information of emotions. The attentional mechanism is calculated using Equation 10.


(10) $$\begin{array}{c} \text{Attention}(Q,K,V)=\text{softmax}(\frac{QK^{T}}{\sqrt{d_{k}}})V \end{array}$$

where Q is the query matrix, K is the key matrix, V is the value matrix, and $d_{k}$ is the dimensionality of the key vectors.

**Figure 9:**
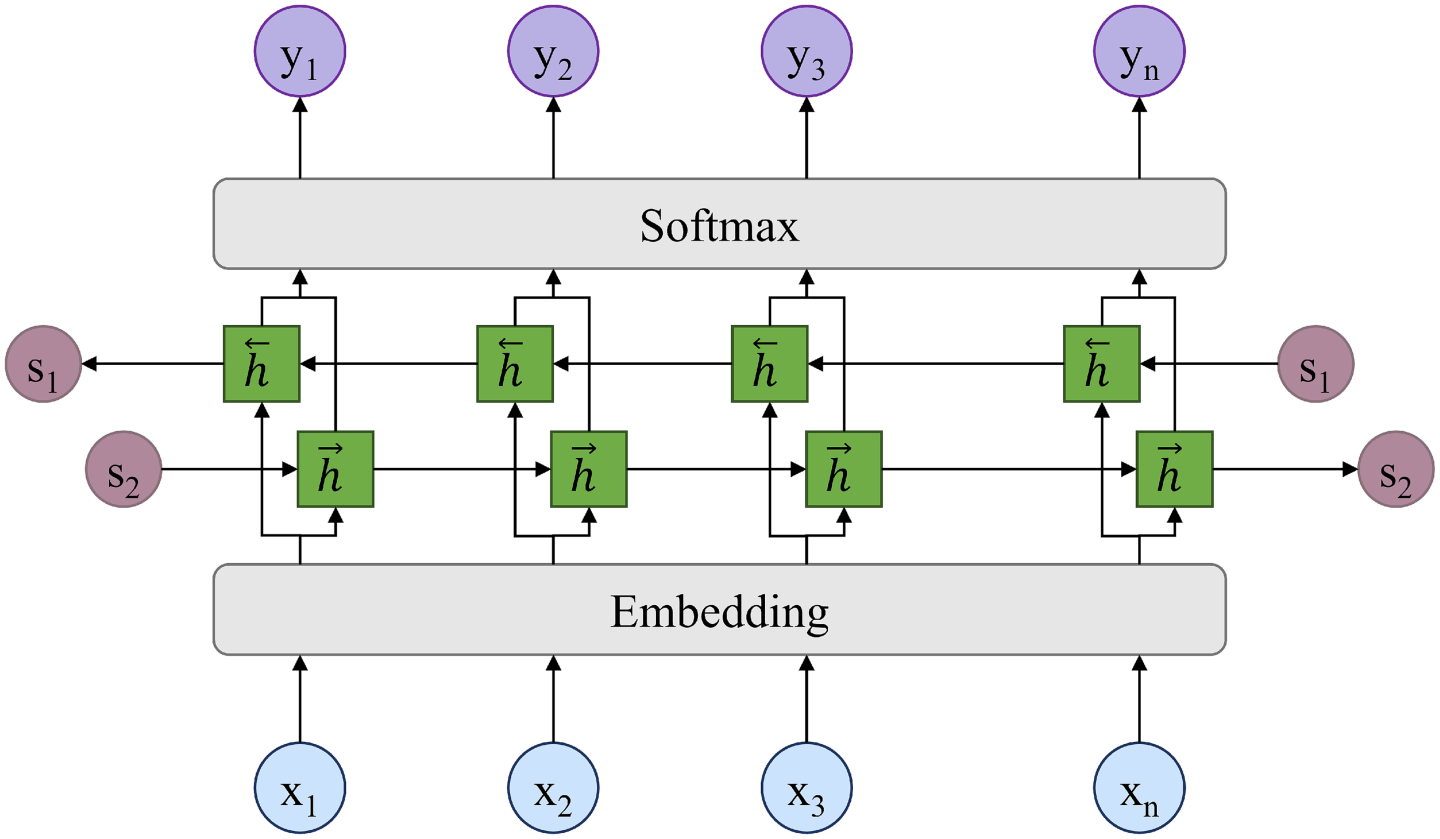
BiLSTM layer.

A long short-term memory neural network is a kind of time-series recurrent neural network. Its network structure consists of one or more units with forgettable and memorable functions. Thus, LSTM is suitable for processing and predicting important events with very long intervals and delays in time series. It was proposed in 1997 to solve the problem of weight disappearance in traditional RNN backpropagation over time. The important components include the forget gate, input gate, and output gate. These gates are responsible for deciding whether the current input is adopted, whether it is remembered in the long term, and whether the input in memory is output in the present. As shown in Figure 9, BiLSTM is a combination of a forward LSTM and a backward LSTM. As a result, it can capture both forward and backward timing information of time series, resulting in improved prediction results. Although the attention mechanism effectively captures global spatio-temporal dependencies, it provides limited explicit modeling of the local sequential continuity that characterizes emotion trajectories. To complement this, a BiLSTM layer is incorporated after the attention module. By processing the sequence bidirectionally, BiLSTM captures fine-grained temporal transitions and preserves contextual continuity. In this architecture, the attention mechanism supplies a global selective focus, while BiLSTM refines local temporal dynamics and reinforces long-range contextual information. Together, they yield a more comprehensive representation of spatio-temporal emotion interactions.

In the final step, we concatenate the feature vectors that represent spatio-temporal emotional dependencies. Next, concatenated vectors are inputted into a fully connected neural network containing a softmax layer to predict the location and corresponding emotional quadrants. The model predicts location and emotion jointly by employing shared layers.

## 4 Case study

### 4.1 Model evaluation

In this study, 80% of the data are taken at random from the sample set for training the model, while the remaining 20% are utilized to validate the model’s correctness. The error is estimated using the cross-entropy loss function in Equation 11.


(11) $$\begin{array}{c} L=-\frac{1}{N}\sum_{i}\sum_{c=1}^{m}y_{\mathrm{ic}}\log(p_{\mathrm{ic}}) \end{array}$$

where *M* denotes the number of categories; *y_ic_* is the sign function that returns 1 if the true category of sample *i* is equal to *c* and 0 otherwise. $p_{\mathrm{ic}}$ is the predicted probability that the observed sample belongs to category *c.*

On this basis, we utilize *k*-fold cross-validation to divide the training set into *k* subsamples, maintaining one subsample for model validation and using the remaining *k-*1 samples for training. Cross-validation is performed *k* times, once for each subsample, and the results are then averaged *k* times to obtain a single estimate.

Training was performed on an Nvidia 4,060 GPU, requiring approximately 4 h for 256 epochs with a batch size of 64. The model contains approximately 470 K parameters, which is relatively lightweight compared to deep learning architectures typically used in this domain.

### 4.2 Analysis of the results

**Figure 10:**
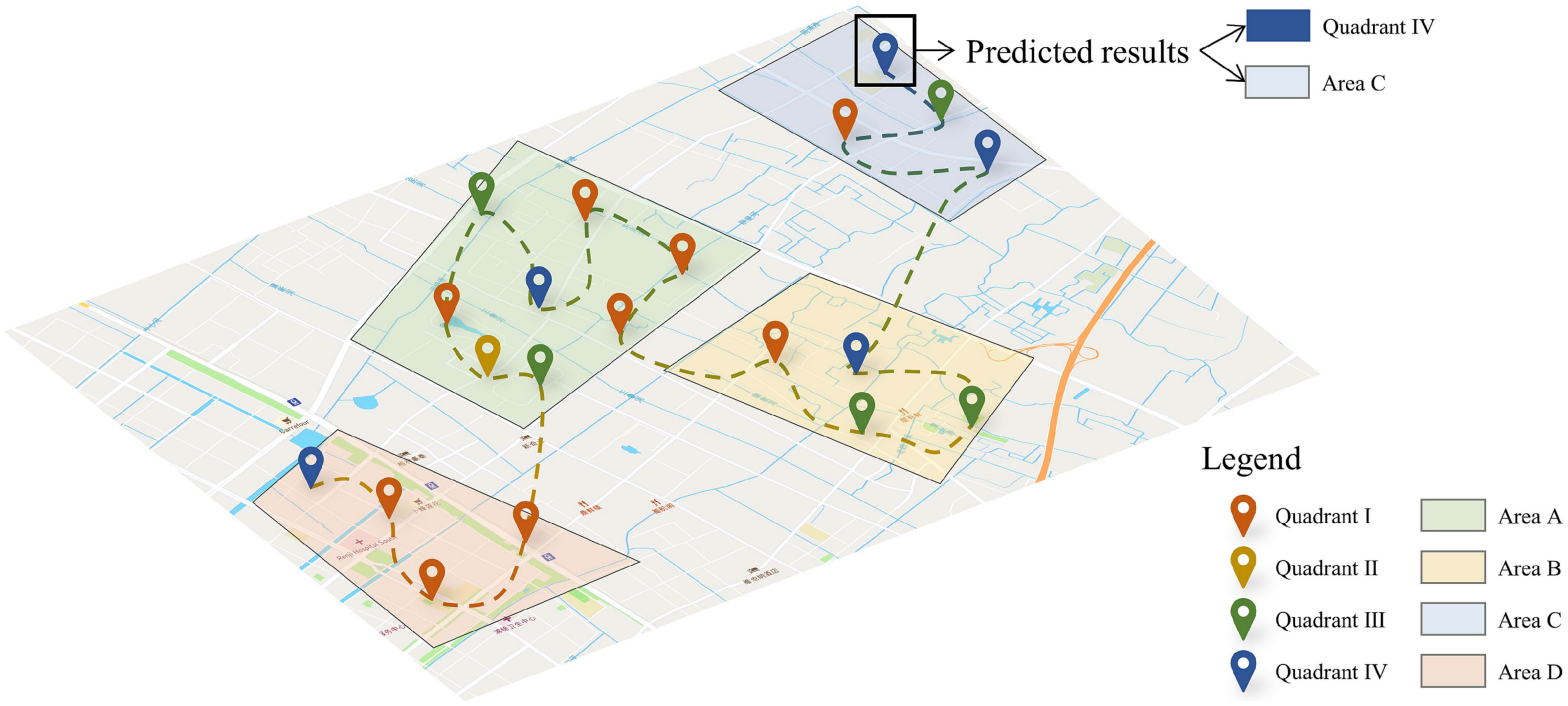
Spatial–temporal prediction of user emotions.

In this section, to present our prediction results in a more visual and detailed way, we selected one user and plotted his check-in locations and emotional status in different clustering areas. As shown in Figure 10, users’ check-in locations are distributed in Areas A, B, C and D. Then, we will analyze the prediction accuracy of location emotions from three perspectives: clustering scale, emotional quadrant, and sequence length. By capturing and constructing the multidimensional spatio-temporal dependencies of users’ check-in location and emotional status, the proposed model achieves high-precision prediction in location emotion and helps analyze the spatio-temporal pattern of users’ emotional states.

The association between prediction accuracy, precision, recall and F1 score are shown in Tables 2–5. Compared with single models, our method has the advantage for all numbers of clustered areas. When the number of clustered areas is 100, the prediction accuracy of our proposed model is 75.21%. The accuracies of the ANN, CNN, BiLSTM, ResNet and ConvBiLSTM are 69.95, 73.81, 73.37, 71.76 and 74.27%, respectively. Our model accuracy has improved by 5.26, 1.40, 1.84, 3.45 and 0.94%. In addition to accuracy, our method also demonstrates consistent improvements in precision, recall and F1 score. Even when the number of clustered areas reaches 500, our method still has a prediction accuracy of 69.49%. In addition to accuracy, our method also demonstrates consistent improvements in precision, recall and F1 score.

**Table 2:** Prediction performance comparison with baselines (accuracy).

Models	Number of clustered areas				
	100	200	300	400	500
ANN	0.6995	0.6563	0.6531	0.6392	0.6209
CNN	0.7381	0.6911	0.6647	0.6511	0.6492
BiLSTM	0.7337	0.6904	0.6773	0.6569	0.6636
ResNet	0.7176	0.6824	0.6619	0.6418	0.6314
ConvBiLSTM	0.7427	0.7161	0.6963	0.6915	0.6853
**Attention-Based BiLSTM**	**0.7521**	**0.7212**	**0.7208**	**0.7196**	**0.6949**

The bold values indicate the prediction performance of the model proposed in this study.

**Table 3:** Prediction performance comparison with baselines (precision).

Models	Number of clustered areas				
	100	200	300	400	500
ANN	0.6895	0.6469	0.6386	0.6261	0.6120
CNN	0.7166	0.6741	0.6635	0.6529	0.6343
BiLSTM	0.7298	0.6834	0.6644	0.6547	0.6419
ResNet	0.7074	0.6634	0.6502	0.6456	0.6228
ConvBiLSTM	0.7283	0.6952	0.6729	0.6635	0.6587
**Attention-Based BiLSTM**	**0.7423**	**0.7118**	**0.7072**	**0.6923**	**0.6859**

The bold values indicate the prediction performance of the model proposed in this study.

**Table 4:** Prediction performance comparison with baselines (recall).

Models	Number of clustered areas				
	100	200	300	400	500
ANN	0.6824	0.6403	0.6273	0.6156	0.6058
CNN	0.7092	0.6671	0.6463	0.6391	0.6277
BiLSTM	0.7181	0.6723	0.6573	0.6456	0.6316
ResNet	0.7049	0.6515	0.6363	0.6219	0.6116
ConvBiLSTM	0.7211	0.6884	0.6773	0.6663	0.6522
**Attention-Based BiLSTM**	**0.7312**	**0.7012**	**0.6978**	**0.6887**	**0.6756**

The bold values indicate the prediction performance of the model proposed in this study.

**Table 5:** Prediction performance comparison with baselines (F1 score).

Models	Number of clustered areas				
	100	200	300	400	500
ANN	0.6859	0.6436	0.6329	0.6208	0.6089
CNN	0.7129	0.6706	0.6548	0.6459	0.6310
BiLSTM	0.7239	0.6778	0.6608	0.6501	0.6367
ResNet	0.7061	0.6574	0.6432	0.6335	0.6171
ConvBiLSTM	0.7247	0.6918	0.6751	0.6649	0.6554
**Attention-Based BiLSTM**	**0.7367**	**0.7065**	**0.7025**	**0.6905**	**0.6807**

The bold values indicate the prediction performance of the model proposed in this study.

In terms of emotional quadrants, we compared the location prediction accuracy of the model in the four emotion quadrants and for different clustering scales. The prediction results are shown in Tables 6A–E. The four quadrants here represent four emotional states with significant emotional differences. According to the Russell ring model, Quadrants I and IV represent positive emotional states, and Quadrants II and III represent negative emotional states. The prediction accuracy of our model is better than that of existing models in all emotional quadrants. In particular, our model has a significant prediction accuracy advantage with regards to positive emotional states (Quadrant I and Quadrant IV). The first quadrant (high valence-high intensity state) had the highest accuracy, followed by the fourth quadrant (high valence-low intensity state), then the third quadrant (low valence-low intensity state), and finally the second quadrant (low valence-high intensity state). This discrepancy is primarily attributable to the imbalanced distribution of emotions in online data. Online users tend to express positive opinions (Yang and Xu, 2023), resulting in a predominance of positive samples in our dataset. Specifically, Quadrants 1 and 4 (positive emotions) account for 73.4% of the data, while Quadrants 2 and 3 (negative emotions) together represent only 26.6%. Within this minority group, Quadrant 2 accounts for merely 12.7%. Such class imbalance restricts the model’s exposure to negative samples during training, leading to insufficient learning of negative emotion features. Consequently, the model achieves higher prediction accuracy for Quadrants 1 and 4, while accuracy for Quadrant 2 is considerably lower.

**Table 6:** Prediction accuracy for four emotional quadrants.

(a)					
Models	Number of clustered areas	100			
	Emotional quadrant	I	II	III	IV
ANN		0.7128	0.6628	0.6703	0.6998
CNN		0.7549	0.7035	0.7029	0.7404
BiLSTM		0.7514	0.7013	0.6992	0.7369
ResNet		0.7447	0.6883	0.6859	0.7271
ConvBiLSTM		0.7699	0.7176	0.7189	0.7551
**Attention-Based BiLSTM**		**0.7709**	**0.7154**	**0.7245**	**0.7683**

(b)					
Models	Number of clustered areas	200			
	Emotional quadrant	I	II	III	IV
ANN		0.7102	0.6527	0.6648	0.6988
CNN		0.7434	0.7014	0.6994	0.7498
BiLSTM		0.7475	0.7047	0.7027	0.7403
ResNet		0.7266	0.6818	0.6881	0.7272
ConvBiLSTM		0.7536	0.7015	0.7055	0.7489
**Attention-Based BiLSTM**		**0.7594**	**0.7065**	**0.7117**	**0.7519**

(c)					
Models	Number of clustered areas	300			
	Emotional quadrant	I	II	III	IV
ANN		0.6888	0.6365	0.6427	0.6907
CNN		0.7305	0.6795	0.6794	0.7276
BiLSTM		0.7288	0.6801	0.6883	0.7354
ResNet		0.7094	0.6557	0.6543	0.7059
ConvBiLSTM		0.7461	0.6815	0.6865	0.7339
**Attention-Based BiLSTM**		**0.7539**	**0.6895**	**0.6996**	**0.7422**

(d)					
Models	Number of clustered areas	400			
	Emotional quadrant	I	II	III	IV
ANN		0.6888	0.6365	0.6427	0.6907
CNN		0.7305	0.6795	0.6794	0.7276
BiLSTM		0.7288	0.6801	0.6883	0.7354
ResNet		0.7128	0.6596	0.6656	0.7072
ConvBiLSTM		0.7356	0.6816	0.6710	0.7387
**Attention-Based BiLSTM**		**0.7539**	**0.6895**	**0.6996**	**0.7422**

(e)					
Models	Number of clustered areas	500			
	Emotional quadrant	I	II	III	IV
ANN		0.6888	0.6365	0.6427	0.6907
CNN		0.7305	0.6795	0.6794	0.7276
BiLSTM		0.7288	0.6801	0.6883	0.7354
ResNet		0.7176	0.6576	0.6571	0.7025
ConvBiLSTM		0.7422	0.6722	0.6859	0.7395
**Attention-Based BiLSTM**		**0.7539**	**0.6895**	**0.6996**	**0.7422**

The bold values indicate the prediction performance of the model proposed in this study.

**Figure 11:**
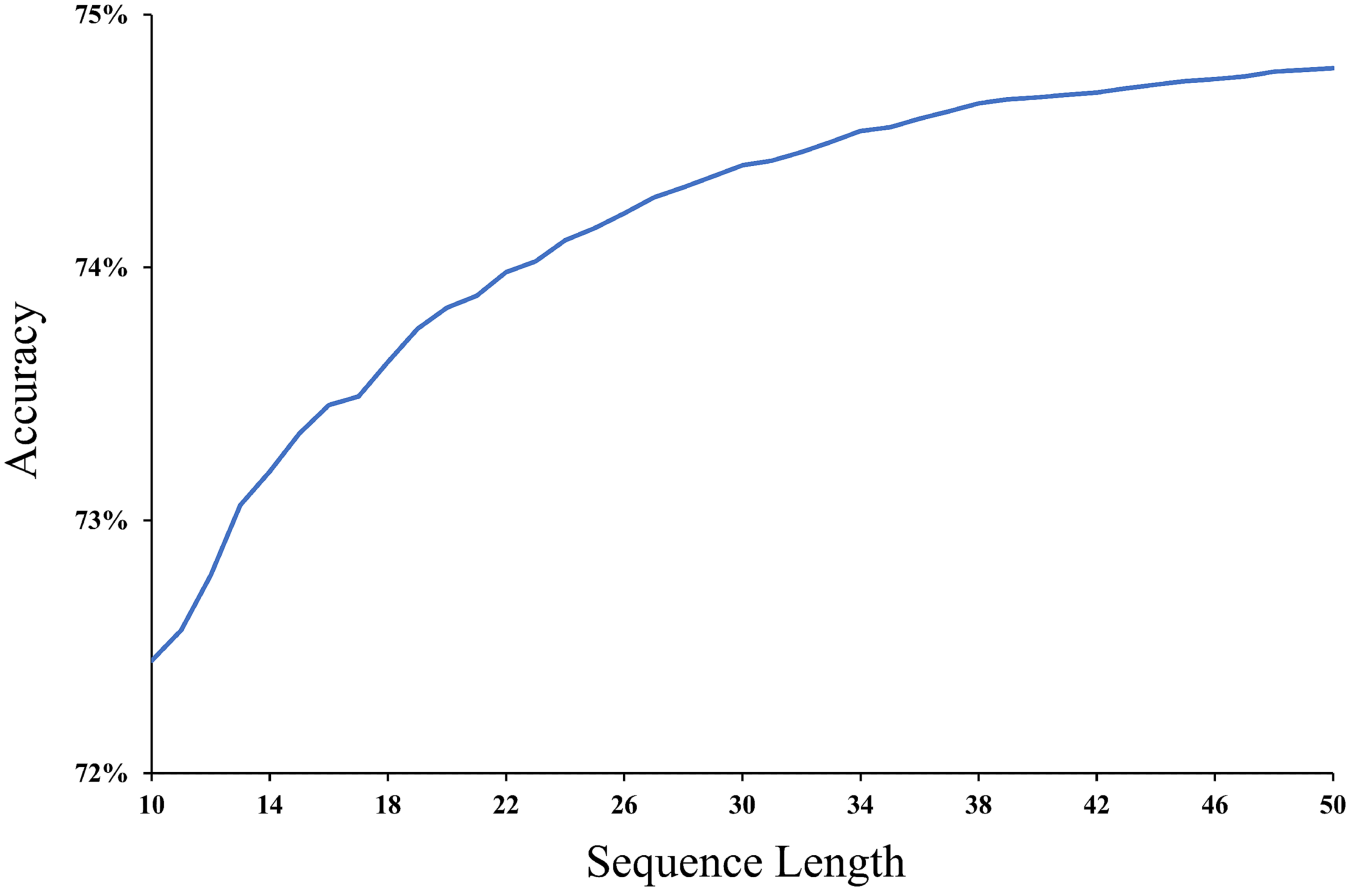
Prediction accuracy for different sequence lengths.

The effect of the sequence length on the accuracy of emotion prediction is shown in Figure 11. In this study, the sequence length refers to the number of user check-in points, and sequence lengths range from 10 to 50. In the dataset employed in this study, the maximum length of a user check-in sequence is up to 51, thereby constraining the influence of sequence length on prediction accuracy to sequences of up to 50. The results of Figure 11 show that the prediction accuracy gradually increases with an increasing sequence length. The accuracy reaches 74.8% after the check-in sequence length reaches 50. The longer the sequence length is, the more spatio-temporal emotional information users’ emotion trajectory provides, which is the basis for constructing spatio-temporal emotional dependencies. By accurately constructing dependencies, our model achieves better prediction results.

## 5 Conclusion and discussion

Spatio-temporal emotion prediction is a major challenge for behavioral and psychological research. It is also important for promoting urban development, boosting the happiness of urban residents, and optimizing the public emotional experience. Existing prediction methods have difficulty effectively constructing multidimensional spatio-temporal emotion dependence. Therefore, human activities and emotional states are difficult to accurately predict. In this case, we propose a new prediction approach coupling an attention mechanism and BiLSTM to predict users’ location emotions.

With the support of the geotagged image data, the proposed method achieves 75.21% accuracy in predicting location emotion. By coupling the attention mechanism with BiLSTM, multidimensional spatio-temporal emotion dependence can be generated. Based on this, the proposed method can not only differentiate four significantly different emotional states but also predict users’ locations with a high precision and effectiveness. Existing research methods can only conduct emotion states or location alone. Compared with these methods, our approach can predict location and emotion at the same time and attach emotional attributes to location-based services. The results provide a novel approach for personalizing emotion-optimized services, enhancing service quality, and demonstrating humanistic care.

The approach in this paper has the following shortcomings. First, the data sources are limited, and more kinds of data, such as text, audio, and physiological electricity, can be introduced in the future. The integration of real-time prediction models will be explored, enabling adaptation to dynamic changes in user location and context. For example, a traffic prediction system could continuously update recommendations based on real-time data, including user location, traffic conditions, and weather patterns. Machine learning techniques, such as reinforcement learning algorithms, will also be employed to refine predictions and ensure more personalized and accurate suggestions for users. Additionally, given the privacy concerns associated with location-based services, differential privacy techniques will be investigated to add noise to location data, protecting user identities. Other privacy-enhancing methods, such as federated learning, will be explored to allow data processing on user devices, minimizing the need to share raw location data with centralized servers. Second, there is a lack of consideration of geographic environmental information. The influence of environmental information on the prediction will be further explored in the future. Environmental factors, such as weather conditions, public events, and surrounding infrastructure, play an important role in location-based predictions. We intend to collect fine-grained environmental data and integrate these elements as node attributes in the model. For instance, weather data (e.g., temperature, precipitation, humidity) could be included as time-dependent features at each node. Event schedules (e.g., concerts, sports events) could be encoded as categorical features associated with event-related nodes. These additions would enhance the model’s ability to account for environmental factors and improve the accuracy of location-based predictions. Third, our model can only distinguish four categories of emotion, and it is difficult to calculate emotion attributes quantitatively. We will further improve the prediction methods, such as predicting the valence and intensity of emotions according to the Russel ring model. In terms of scalability, the dataset used in this work was constrained by the limitations of the Sina Weibo platform, which prevents further data collection^1^. We plan to extend this research by collecting additional data from other social media platforms, such as Twitter, to test the model’s performance on a larger and more diverse dataset. Emotional expressions in online environments may diverge from those in offline contexts. Constrained by the limitations of online platforms and affected by expressive biases, online users are inclined to exaggerate their emotions (Caspi and Etgar, 2023). To mitigate this limitation, we plan to incorporate real-world offline data in future work. Through comparative analysis, we aim to correct the potential biases present in online datasets.

## Data Availability

The raw data supporting the conclusions of this article will be made available by the authors, without undue reservation.

## Footnotes

^1^ https://open.weibo.com/wiki/
